# Fat and Lean

**Published:** 1883-10

**Authors:** 


### FAT AND LEAN.

THERE are two questions that we are called upon to answer more frequently than any others: one is, what shall I do to reduce my weight, without impairing my health? The other is the directly opposite question. The first proposition is susceptible of a solution, or at least it would be, were it possible to find a fleshy person who would submit himself for a sufficient time to the observance of a few simple rules. I have never yet met such a patient, and have not therefore had opportunity to test any plan of treatment. Some years ago I became acquainted, in South America, with the captain of an English steamship. He was a very large man, being over six feet high, and weighed 360 pounds. Our acquaintance continued for two years, and then I did not see him again for about a year, when one day we found ourselves seated opposite to each other at the table on the steamer "Northern Light." He called me by name, and asked me if I recognized him, but I did not. When he told me his name I could trace some resemblance to his former self; but a reduction in weight from 360 to 180 pounds had almost blotted out his identity. I became greatly interested in the case, and he seemed pleased to give me all the information I required on the subject. What was known as the "Banting System" was then much talked about, and it was to the practice of this system that the reducing process was due. Without going into details, it may be stated that the Banting system consisted in abstaining almost entirely from fats and fat-producing foods. The captain said he was in better health than when loaded with such a weight of flesh.

The simple rule to reduce fat is to reduce the supply of those foods that make fat; conspicuous among which are fat meats, butter, milk, potatoes, and bread made of fine wheat flour.

But to do the other thing? aye, there's the rub. How can we force the stomach of a lean man to digest more food than it craves or the system to store it up? Much may be done where the conditions are favorable. If a person is so situated that the cares of life do not weigh heavily upon him; if he is cheerful, and of a happy disposition, and in good health, and—and—well, we give up; such persons are always plump, anyway. "Laugh and grow fat" is a terse way of stating a well-recognized fact.

Leanness is said to be constitutional; it would be near the mark to say that, as a rule, it results from nervous irritability, either acquired or inherited. If this disposition could be changed, so that the ordinary wear and tear of existence did not fret and worry the individual, he would have taken a long step in the direction of happiness and *plumptitude.*

